# Longitudinal trajectories of a claims-based frailty measure during adjuvant chemotherapy in women with stage I-III breast cancer

**DOI:** 10.1093/oncolo/oyae092

**Published:** 2024-05-08

**Authors:** Emilie D Duchesneau, Katherine Reeder-Hayes, Til Stürmer, Dae Hyun Kim, Jessie K Edwards, Jennifer L Lund

**Affiliations:** Department of Epidemiology and Prevention, Division of Public Health Sciences, Wake Forest University School of Medicine, Winston-Salem, NC; Lineberger Comprehensive Cancer Center, University of North Carolina at Chapel Hill, Chapel Hill, NC; Division of Oncology, Department of Medicine, University of North Carolina at Chapel Hill, Chapel Hill, NC; Lineberger Comprehensive Cancer Center, University of North Carolina at Chapel Hill, Chapel Hill, NC; Department of Epidemiology, Gillings School of Global Public Health, University of North Carolina at Chapel Hill, Chapel Hill, NC; Marcus Institute for Aging Research, Hebrew SeniorLife, Harvard Medical School, Boston, MA; Division of Gerontology, Department of Medicine, Beth Israel Deaconess Medical Center, Boston, MA; Department of Epidemiology, Gillings School of Global Public Health, University of North Carolina at Chapel Hill, Chapel Hill, NC; Lineberger Comprehensive Cancer Center, University of North Carolina at Chapel Hill, Chapel Hill, NC; Department of Epidemiology, Gillings School of Global Public Health, University of North Carolina at Chapel Hill, Chapel Hill, NC

**Keywords:** breast cancer, adjuvant chemotherapy, frailty, trajectory analysis, claims data, SEER-Medicare

## Abstract

**Background:**

Frailty is a dynamic syndrome characterized by reduced physiological reserve to maintain homeostasis. Prospective studies have reported frailty worsening in women with breast cancer during chemotherapy, with improvements following treatment. We evaluated whether the Faurot frailty index, a validated claims-based frailty measure, could identify changes in frailty during chemotherapy treatment and identified predictors of trajectory patterns.

**Methods:**

We included women (65+ years) with stage I-III breast cancer undergoing adjuvant chemotherapy in the SEER-Medicare database (2003-2019). We estimated the Faurot frailty index (range: 0-1; higher scores indicate greater frailty) at chemotherapy initiation, 4 months postinitiation, and 10 months postinitiation. Changes in frailty were compared to a matched noncancer comparator cohort. We identified patterns of frailty trajectories during the year following chemotherapy initiation using K-means clustering.

**Results:**

Twenty-one thousand five hundred and ninety-nine women initiated adjuvant chemotherapy. Mean claims-based frailty increased from 0.037 at initiation to 0.055 4 months postchemotherapy initiation and fell to 0.049 10 months postinitiation. Noncancer comparators experienced a small increase in claims-based frailty over time (0.055-0.062). We identified 6 trajectory patterns: a robust group (78%), 2 resilient groups (16%), and 3 nonresilient groups (6%). Black women and women with claims for home hospital beds, wheelchairs, and Parkinson’s disease were more likely to experience nonresilient trajectories.

**Conclusions:**

We observed changes in a claims-based frailty index during chemotherapy that are consistent with prior studies using clinical measures of frailty and identified predictors of nonresilient frailty trajectories. Our study demonstrates the feasibility of using claims-based frailty indices to assess changes in frailty during cancer treatment.

## Teaser

Prospective studies have reported frailty worsening in older women with breast cancer during chemotherapy, with improvements following treatment. This study evaluated whether the Faurot frailty index, a validated claims-based frailty measure, could identify changes in frailty during adjuvant chemotherapy treatment.

**Implications for Practice**

Using a claims-based frailty proxy measure (the Faurot frailty index), we found that women with early-stage breast cancer experienced an increase in frailty during adjuvant chemotherapy, peaking at 4 months postinitiation and declining by 10 months postinitiation. Six percent of women receiving adjuvant chemotherapy exhibited nonresilient frailty trajectories, with factors including older age, Black race, and having baseline claims for durable medical equipment being associated with nonresilient trajectories.

## Introduction

Approximately half of patients with breast cancer are diagnosed during older age (≥65 years).^1,2^ Frailty is a dynamic aging-related syndrome characterized by reduced physiologic reserve to maintain homeostasis.^3-5^ Frail individuals with breast cancer are more likely to report high-grade treatment toxicities, discontinue treatment, or die than their robust counterparts.^6,7^

Resilience, a concept related to frailty, refers to the ability to bounce back or recover from a stressor.^8,9^ While many patients with cancer experience short-term frailty worsening during treatment, resilient patients recover from these effects following treatment completion. Prior prospective studies have shown that many older women with breast cancer experience worsening in frailty during chemotherapy, with longer-term improvements 6 months to 1 year following the end of treatment.^10-13^ Identifying older adults with cancer who are at risk for frailty progression is critical for treatment decision-making and for targeting interventions aimed at preventing or reversing frailty, such as nutritional or physical activity interventions.^14^

Characterizing changes in frailty and resilience in cancer survivors is challenging using the tools available for measuring frailty. While comprehensive geriatric assessment is the gold standard for assessing frailty in older adults with cancer, it is difficult to implement and underutilized in routine oncology practice and large-scale research settings.^15^ Medicare claims and enrollment data, with linkage to the Surveillance, Epidemiology, and End Results (SEER) cancer registries, may offer a solution for measuring changes in frailty over time as they continuously capture health care encounter information for large and diverse cancer populations.^16^

The Faurot frailty index is a Medicare claims-based proxy measure that predicts frailty in older adults using demographic, diagnostic, procedural, and durable medical equipment billing information.^17-19^ It has previously been used to identify frail cancer survivors and to address confounding by frailty in studies using SEER-Medicare data.^20-22^ However, the ability of the Faurot frailty index to identify longitudinal changes in frailty during cancer treatment has not been assessed. We assessed whether changes in claims-based frailty during adjuvant chemotherapy treatment in women with early-stage breast cancer were consistent with patterns observed in prior research using a clinical frailty measure.^10,11^ We also identified patterns of claims-based frailty trajectories during the year following adjuvant chemotherapy initiation and identified risk factors for experiencing frailty worsening without signs of resilience.

## Materials and methods

The Office of Human Research Ethics of the University of North Carolina at Chapel Hill approved this study (22-0115). Analyses were performed using SAS Version 9.4 (SAS Institute, Cary, NC).

### Data source

We used SEER-Medicare data from 2003 to 2019. SEER is a collection of population-based cancer registries in the United States that collect information on tumor, demographic, and cause of death information for persons with incident cancers residing in the SEER regions.^23^ SEER has been linked to Medicare claims and enrollment data through a collaboration between the National Cancer Institute and the Centers for Medicare & Medicaid Services. SEER-Medicare includes a 5% sample of Medicare beneficiaries without cancer residing in the SEER regions.

### Study population

**Figure 1.**
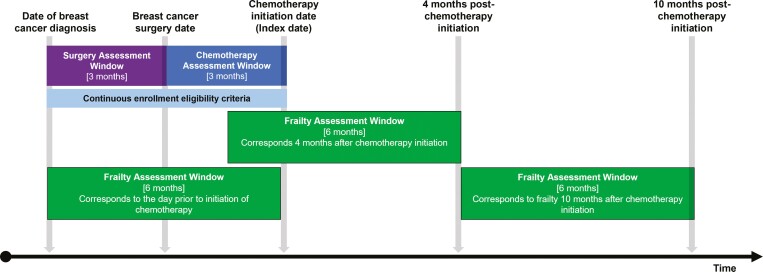
Study schematic for benchmark analysis. For inclusion, women were required to undergo breast surgery within 3 months (90 days) of diagnosis and to initiate chemotherapy within 3 months (90 days) of surgery. Claims-based frailty was assessed at 3 timepoints (chemotherapy initiation, 4 months postchemotherapy initiation, and 10 months postchemotherapy initiation) using 6-month (180-day) frailty ascertainment windows. Abbreviations: BC, breast cancer; HMO, health maintenance organization; NAC, neoadjuvant chemotherapy.

We identified older women (≥65 years) diagnosed with American Joint Committee on Cancer (AJCC) stage I-III breast cancer between 2004 and 2017 (AJCC 6th edition for those diagnosed between 2004 and 2010 and 7th edition for those diagnosed after 2010). We required women to have undergone mastectomy or breast-conserving surgery within 90 days of diagnosis and to have commenced adjuvant chemotherapy within 90 days after surgery (Figure 1). The date of chemotherapy initiation was the index date for analysis. We required continuous enrollment in Medicare fee-for-service between the date of cancer diagnosis and the index date or for at least 180 days prior to the index date (whichever was longer). Women who received neoadjuvant chemotherapy and those who did not have primary surgery were excluded. Codes to identify study variables are provided in Supplementary Table S1.

### Noncancer comparators

We constructed a matched comparator cohort of women without cancer from the SEER-Medicare 5% noncancer sample. Each woman in the adjuvant chemotherapy cohort was matched to up to 5 female Medicare beneficiaries without cancer using exact matching on year of birth and SEER region. Matching was done randomly with replacement. Matched noncancer comparators were assigned the index date of their matched woman with breast cancer and were required to meet the same continuous enrollment eligibility requirements.

### Claims-based frailty

We calculated claims-based frailty using the Faurot frailty index, a Medicare claims-based proxy measure based on demographic, enrollment, and diagnostic, procedural, and durable medical equipment billing information.^17,18^ The Faurot frailty index was initially developed and validated as a predictor of disability in the activities of daily living.^17^ It was externally validated in both the Atherosclerosis Risk in Communities and National Health and Aging Trends Study cohorts as a predictor of the frailty phenotype.^19,24^ Among older women with breast cancer in the SEER-Medicare database, the Faurot frailty index was shown to be strongly predictive of 1-year outcomes relevant to frail populations, including mortality, skilled nursing facility admissions, and hospitalizations.^25^ The components of the Faurot frailty index include indicators associated with lower likelihood of frailty (eg, lipid screening and cancer screening), and indicators associated with greater frailty (eg, wheelchairs). The measure ranges from 0 to 1, with a higher score indicating a higher probability of being frail. A full description of the index has been published previously.^17,18^ In the primary analysis, claims-based frailty was calculated using a 180-day frailty ascertainment window prior to the timepoint of interest (Figure 1).^26^

### Covariates

We assessed baseline and time-varying covariates to account for differences in covariate distributions between the adjuvant chemotherapy and comparator cohorts and to address potentially informative attrition. Demographic variables included age, racial and ethnic category (Alaska Native or American Indian, Asian or Pacific Islander, Black, Hispanic, White), and region. Cancer characteristics included year of diagnosis, stage, grade, and subtype. Time-varying comorbidities were assessed using the Gagne comorbidity index using claims from the 180 days prior to each follow-up timepoint.^27,28^ A proxy for overall health status included receipt of a flu vaccination during the 180 days prior to the index date.^29,30^

### Benchmark analysis

We followed women undergoing adjuvant chemotherapy to identify changes in claims-based frailty at 3 timepoints (Figure 1)^26^: on the date of chemotherapy initiation, 4 months postinitiation, and 10 months postinitiation. Frailty at each timepoint was assessed using claims during the 180 days prior to the timepoint. Four months postinitiation was selected to reflect the period near the end of adjuvant chemotherapy treatment, which typically ranges from 3 to 6 months. Ten months postinitiation reflects the period approximately 6 months after the end of chemotherapy completion when we hypothesized that some of the detrimental effects of chemotherapy on frailty may have reversed. These timepoints were similar to those used in a prospective observational study that served as the benchmark for our analysis.^10^

We compared changes in the Faurot frailty index for women undergoing adjuvant chemotherapy to changes in the comparator cohort using generalized estimating equations (GEE) with a gamma distribution, identity link, and within-subject autoregressive correlation structure.^31^ Changes in claims-based frailty over time were assessed by including indicators for time and interactions between time and cohort (ie, cancer or comparator cohort).

We addressed differences in baseline characteristics between the adjuvant chemotherapy and noncancer comparator cohort by standardizing the covariate distribution of the noncancer comparator cohort to reflect the distribution in the adjuvant chemotherapy cohort via standardized mortality ratio (SMR) weighting.^32^ We used inverse probability of attrition weighting to address potentially informative disenrollment from Medicare fee-for-service or death that occurred between follow-up timepoints.^33^ This approach upweights individuals who remain under observation in a study to stand in for those who disenroll or die. Separate pooled logistic regression models were fit for each type of attrition that included baseline demographics and time-varying comorbidity and frailty information.

#### Sensitivity analyses

We conducted 2 sensitivity analyses to assess whether changes in the claims-based frailty score were primarily driven by changes in health services. First, we conducted a subgroup analysis in women who received a breast cancer screening mammogram during the year prior to diagnosis. The comparator cohort in this analysis included women in the 5% noncancer sample who received a screening mammogram with no subsequent breast cancer diagnosis. In the second sensitivity analysis, we removed the indicators for cancer and lipid screening from the calculation of the Faurot frailty index. Therefore, any changes in the claims-based frailty score over time were driven by changes in the other frailty indicators.

### Trajectory analysis

We conducted an exploratory analysis to identify patterns of claims-based frailty trajectories during the year following chemotherapy initiation. We calculated the predicted probability of frailty at 30-day intervals for 12 months using 180-day frailty ascertainment windows starting from the date of adjuvant chemotherapy initiation (Supplementary Figure S1).

We used K-means longitudinal clustering to identify distinct trajectory patterns.^34,35^ K-means longitudinal clustering is an iterative, nonparametric method that classifies individuals into trajectory clusters by maximizing between-cluster separation while minimizing within-cluster variation. This algorithm assigns each woman to a trajectory cluster with the nearest multivariate mean, iterating until the cluster assignments stabilize. Individuals were required to have at least 3 months of follow-up and contributed to all months prior to disenrollment or death. We allowed for 2-8 clusters and determined the optimal number of clusters using statistical criteria, specifically the Genolini variant of the Calinski-Harabasz criterion.^36^ We identified demographic and clinical predictors of experiencing nonresilient frailty trajectories using a logistic regression model.

## Results

### Cohort characteristics

**Figure 2.**
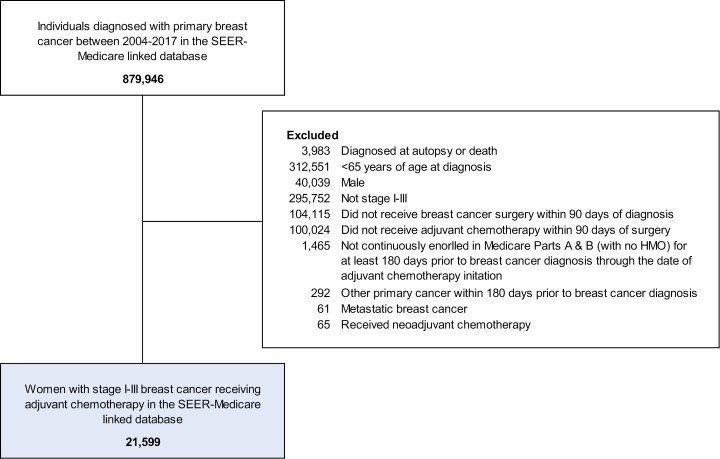
Consort diagram. Abbreviations: HMO, health maintenance organization; SEER, Surveillance, Epidemiology, and End Results.

We identified 21 599 women with early-stage breast cancer who received adjuvant chemotherapy (Figure 2). The median time between breast surgery and adjuvant chemotherapy initiation was 47 days (IQR 35-61). Characteristics of the study sample and comparator cohort are presented in Table 1. The median age was 70 years (IQR 67-74). Eighty-two percent of women were White, 9% were Black, 6% were Hispanic, 4% were Asian or Pacific Islander, and <1% were American Indian or Alaskan Native. Twenty-two percent of women had stage I breast cancer at diagnosis, 54% had stage II, and 24% had stage III. The distribution of covariates between the adjuvant chemotherapy cohort and the noncancer comparator cohort was well-balanced after SMR weighting.

**Table 1.** Baseline characteristics of women with stage I-III breast cancer receiving adjuvant chemotherapy and noncancer comparators in the SEER-Medicare database.

Characteristic	Adjuvant chemotherapy cohort *N* = 21 599	General noncancer cohort *N* = 107 995	General noncancer cohort (after SMR weighting)
Age, median (IQR)	70 (67, 74)	70 (67, 74)	70 (67, 74)
Race, *n* (%)			
Non-Hispanic White	17 380 (81.6)	80 613 (75.9)	17 385 (81.6)
Black	1889 (8.9)	9915 (9.3)	1888 (8.9)
API	787 (3.7)	6452 (6.1)	1173 (5.5)
Hispanic	1174 (5.5)	8778 (8.3)	786 (3.7)
AI/AN	63 (0.3)	407 (0.4)	63 (0.3)
Missing	306	1830	0
Census region, *n* (%)			
Northeast	4201 (19.4)	21 005 (19.4)	4150 (19.5)
West	8979 (41.6)	44 895 (41.6)	8772 (41.2)
Midwest	4126 (19.1)	20 630 (19.1)	4101 (19.3)
South	4293 (19.9)	21 465 (19.9)	4271 (20.1)
Stage at diagnosis, *n* (%)			
I	4826 (22.3)	N/A	N/A
II	11 594 (53.7)		
III	5179 (24.0)		
Type of surgery, *n* (%)			
Mastectomy	8659 (40.1)	N/A	N/A
BCT	12 940 (59.9)		
T stage, *n* (%)			
T0	38 (0.2)	N/A	N/A
T1	8964 (41.6)		
T2	10 321 (47.9)		
T3	1552 (7.2)		
T4	668 (3.1)		
Missing	56		
N stage, *n* (%)			
N0	9410 (43.6)	N/A	N/A
N1	7835 (36.3)		
N2	2805 (13.0)		
N3	1524 (7.1)		
Missing	25		
Tumor grade, *n* (%)			
Well differentiated	1799 (8.6)	N/A	N/A
Moderately differentiated	8202 (39.1)		
Poorly differentiated	10 815 (51.6)		
Undifferentiated	157 (0.7)		
Missing	626		
Subtype,^a^*n* (%)			
HR+/HER2+	2274 (17.9)	N/A	N/A
HR+/HER2−	6573 (51.7)		
HR−/HER2+	977 (7.7)		
HR−/HER2−	2880 (22.7)		
Missing	8895		
Gagne combined comorbidity score,^b^*n* (%)			
<0	6096 (28.2)	23 901 (22.1)	6034 (28.3)
0	9690 (44.9)	45 752 (42.4)	9514 (44.7)
1	3053 (14.1)	15 938 (14.8)	3017 (14.2)
2	1278 (5.9)	8433 (7.8)	1268 (6.0)
≥3	1482 (6.9)	13 971 (12.9)	1461 (6.9)
Preindex flu vaccine, *n* (%)	6570 (30.4)	25 943 (24.0)	6478 (30.4)

^a^HER2 status is only reliably captured in SEER-Medicare after 2010.

^b^Cancer and metastatic cancer were excluded when calculating the Gagne combined comorbidity score.

Abbreviations: AI/AN, American Indian or Alaska Native; API, Asian or Pacific Islander; BCT, breast-conserving therapy; HER2, human epidermal growth factor receptor 2; HR, hormone receptor; IQR, interquartile range; SMR, standardized morbidity ratio.

### Benchmark analysis results

**Figure 3.**
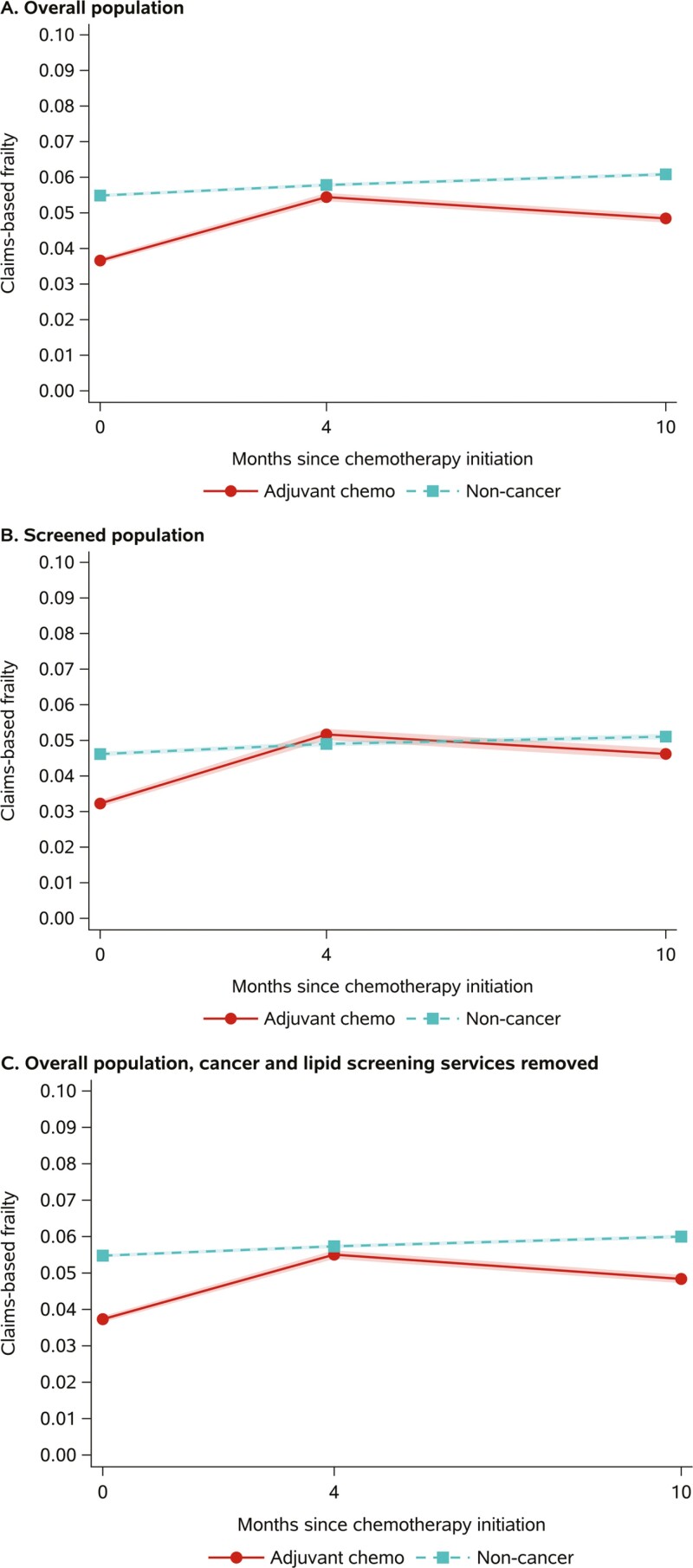
Changes in claims-based frailty in women with breast cancer undergoing adjuvant chemotherapy and noncancer comparators after SMR and inverse probability of attrition weighting. Bands reflect 95% CIs around means. Abbreviation: SMR, standardized mortality ratio.

Three percent of the women receiving adjuvant chemotherapy and 1% of the noncancer comparator cohort died during the 10 months following chemotherapy initiation. Longitudinal changes in claims-based frailty after SMR and inverse probability of attrition weighting are presented in Figure 3A. Women receiving adjuvant chemotherapy experienced slight increases in mean claims-based frailty between the index date and 4 months postindex (0.037-0.055), followed by a decrease 10 months postindex (0.049). Alternatively, the noncancer comparator cohort experienced a slight increase in mean claims-based frailty over time (index: 0.055; 4 months postindex: 0.058; 10 months postindex: 0.062).

The components of the Faurot frailty index with the largest absolute change in prevalence between the index date and 4 months postindex among the adjuvant chemotherapy cohort were cancer screening (57%-14%), lipid abnormalities (64%-55%) and ambulance/life support (4%-10%). The relative changes in the prevalence of hypotension/shock, wheelchairs, decubitus ulcers, and weakness were also large (Supplementary Table S2). The frailty components with the largest change in prevalence between 4 and 10 months postindex were cancer screening (14%-25%), psychiatric diagnoses (25%-18%), and heart failure (19%-13%). Similar results were observed in the subset of individuals who received preindex breast cancer screening (Figure 3B) and in the analysis with cancer and lipid screening services removed as indicators when calculating the Faurot frailty index (Figure 3C).

Results from the GEE models indicated that both the adjuvant chemotherapy and comparator cohorts experienced increases in claims-based frailty over the 2 time periods (Table 2). However, women receiving adjuvant chemotherapy had a larger increase in claims-based frailty between the index date and 4 months postindex (interaction term β=0.014, 95% CI, 0.013 to 0.015), followed by a decrease at 10 months (interaction term β=0.004, 95% CI, 0.003 to 0.005).

**Table 2.** Comparison of changes in claims-based frailty for adjuvant chemotherapy and comparator cohorts over time using standardized mortality ratio and inverse probability of attrition weighted generalized estimating equations.

	Overall	Screened population
	β (95% CI)	β (95% CI)
Intercept	0.055 (0.054, 0.055)	0.046 (0.045, 0.047)
Cohort		
Adjuvant chemotherapy cohort	−0.017 (−0.018, −0.016)	−0.013 (−0.015, −0.012)
Comparator cohort	Ref	Ref
Time		
T1: index date	Ref	Ref
T2: 4 months postindex	0.003 (0.003, 0.004)	0.003 (0.002, 0.003)
T3: 10 months postindex	0.007 (0.007, 0.008)	0.005 (0.004, 0.006)
Interaction terms		
T1 * adjuvant chemotherapy cohort	Ref	Ref
T2 * adjuvant chemotherapy cohort	0.014 (0.013, 0.015)	0.016 (0.015, 0.018)
T3 * adjuvant chemotherapy cohort	0.004 (0.003, 0.005)	0.008 (0.006, 0.010)

### K-means trajectory results

**Figure 4.**
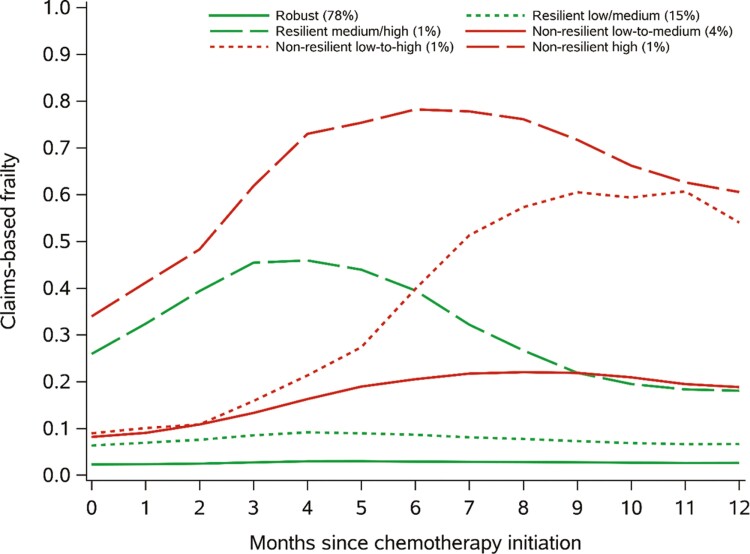
Claims-based frailty trajectories among women receiving adjuvant chemotherapy in the SEER-Medicare database. Abbreviation: SEER, Surveillance, Epidemiology, and End Results.

The K-means clustering algorithm identified 6 trajectory clusters (Figure 4). These clusters included a robust group (78%), a resilient low/medium frailty group (15%), a resilient medium/high frailty group (1%), a nonresilient low-to-medium frailty group (4%), a nonresilient low-to-high frailty group (1%), and a nonresilient high frailty group (1%). The average posterior probabilities of belonging to each cluster were 100%, 89%, 100%, 97%, 97%, and 100%, indicating a good fit of the data.

Baseline characteristics of the adjuvant chemotherapy cohort, stratified by cluster, are presented in Supplementary Table S3. We assessed associations between the baseline characteristics and belonging to a nonresilient frailty trajectory compared to a robust or resilient frailty trajectory using a logistic regression model (Table 3). Black women (OR = 2.34, 95% CI 1.93 to 2.84) and those categorized as Other race (American Indian, Alaskan Native, Asian, or Pacific Islander; OR = 2.09, 95% CI 1.58 to 2.75) were more likely to experience nonresilient trajectories than White women. Women with higher tumor stage and higher comorbidity burden were also more likely to experience nonresilient patterns. The strongest predictors of nonresilient patterns were baseline claims for Parkinson’s Disease (OR = 7.98, 95% CI 4.94 to 12.90), home hospital beds (OR = 5.75, 95% CI 3.12 to 10.59), wheelchairs (OR = 4.19, 95% CI 2.68 to 6.55), and home oxygen (OR = 3.78, 95% CI 2.90 to 4.93). Older age was also associated with an increased risk of nonresilient frailty trajectories (OR = 1.12, 95% CI 1.11 to 1.14).

**Table 3.** Predictors of frailty trajectory patterns in women with stage I-III breast cancer undergoing adjuvant chemotherapy in the SEER-Medicare database.

	OR (95% CI)
Age on the index date (centered at 65 years)	1.12 (1.11, 1.14)
Race (ref: non-Hispanic White)	
Black	2.34 (1.93, 2.84)
Hispanic	0.78 (0.56, 1.09)
Other	2.09 (1.58, 2.75)
Region (ref: West)	
Northeast	0.89 (0.73, 1.09)
Midwest	1.10 (0.91, 1.32)
South	1.16 (0.96, 1.40)
Stage at diagnosis (ref: I)	
II	1.23 (1.00, 1.51)
III	1.57 (1.24, 1.97)
Mastectomy (ref: BCT)	0.88 (0.77, 1.02)
Tumor grade (ref: well differentiated)	
Moderately differentiated	0.99 (0.74, 1.31)
Poorly differentiated	1.30 (0.99, 1.71)
Undifferentiated	1.03 (0.46, 2.30)
Gagne comorbidity score^a^ (ref: ≤0)	
1	1.17 (0.96, 1.43)
2	1.80 (1.45, 2.23)
≥3	2.31 (1.85, 2.89)
Preindex flu vaccine	0.93 (0.80, 1.08)
Components of the Faurot frailty index	
Parkinson’ disease	7.98 (4.94, 12.90)
Home hospital bed	5.75 (3.12, 10.59)
Wheelchair	4.19 (2.68, 6.55)
Home oxygen	3.78 (2.90, 4.93)
Paralysis	2.73 (1.77, 4.20)
Podiatric care	2.11 (1.72, 2.61)
Psychiatric diagnoses	1.93 (1.66, 2.24)
Dementia	1.87 (1.48, 2.37)
Skin ulcer (decubitus)	1.86 (1.28, 2.69)
Stroke/brain injury	1.78 (1.35, 2.35)
Ambulance/life support	1.73 (1.37, 2.18)
Bladder dysfunction	1.70 (1.35, 2.13)
Arthritis/joint conditions	1.44 (1.25, 1.65)
Vertigo	1.22 (0.97, 1.53)
Heart failure	1.18 (0.98, 1.42)
Weakness	1.14 (0.89, 1.48)
Lipid abnormality	0.81 (0.70, 0.94)
Rehabilitation services	0.81 (0.67, 0.97)
Cancer screening	0.79 (0.69, 0.91)
Hypotension/shock	0.48 (0.31, 0.73)

^a^Cancer and metastatic cancer were excluded when calculating the Gagne combined comorbidity score.

Abbreviations: BCT, breast-conserving therapy; OR, odds ratio.

## Discussion

We used SEER-Medicare linked data to estimate changes in frailty over time among older women with stage I-III breast cancer during adjuvant chemotherapy treatment using a claims-based frailty proxy measure. We found that patterns of changes in the mean claims-based frailty score were similar to changes that were observed in prospective studies using clinical measures of frailty.^10,11^ Specifically, women in the adjuvant chemotherapy cohort experienced an increase in claims-based frailty 4 months following chemotherapy initiation (0.037-0.055), followed by improvements 10 months postinitiation (0.049).

Although these average changes are small, prior studies in general populations have shown that older adults with claims-based frailty scores less than 0.05 have lower risks of mortality, hospitalizations, and SNF admissions compared to older adults with claims-based scores between 0.05 and 0.10.^17,18,24^ In addition, a recent validation study found that older adults who experienced a 3-year worsening in the Fried frailty phenotype from robust to prefrail, experienced a simultaneous average change in claims-based frailty from 0.05 to 0.08.^37^ These external validation studies provide further support that the small changes in the average claims-based frailty score we observed in this cohort of women with breast cancer may correspond to clinically meaningful changes in frailty.

We compared changes in claims-based frailty among women receiving adjuvant chemotherapy to a comparator cohort without cancer. Our choice of comparator served several purposes. First, we selected a comparator cohort that closely reflected the comparator cohort studied in Magnuson et al, which served as the benchmark for our analysis. Second, the inclusion of a comparator cohort without cancer allowed us to demonstrate that the changes we observed among women initiating chemotherapy likely reflected changes due to breast cancer diagnosis and treatment, rather than changes due to the aging process. Finally, we opted not to compare across breast cancer treatment strategies since cancer treatment is highly selective and considers numerous patient characteristics that are challenging to fully account for in claims data. Future research should evaluate frailty trajectories among women with breast cancer who receive different treatment strategies (eg, neoadjuvant chemotherapy and hormonal therapy).

We identified patterns of claims-based frailty trajectories using K-means longitudinal clustering, which is an exploratory and hypothesis-generating approach. While most women (78%) had a robust trajectory with minimal changes throughout follow-up, we also identified 2 resilient and 3 nonresilient groups. Resilient patterns exhibited an increase in claims-based frailty during the first 4 months following chemotherapy initiation, followed by decreases that returned claims-based frailty to near baseline levels. Three clusters of women (6%) exhibited nonresilient claims-based frailty trajectory patterns. The first 2 clusters started at low claims-based frailty scores and experienced a sharp increase in their frailty scores during the year following chemotherapy initiation. The third nonresilient cluster had high claims-based frailty at initiation, but still experienced an overall increase in claims-based frailty throughout follow-up.

There were significant racial disparities associated with nonresilient frailty trajectories, with Black women being more likely to experience nonresilient trajectories. Prior research has shown that Black women with breast cancer receive less supportive and survivorship care than White women.^38,39^ Differences in care in addition to differences in the distribution of social determinants of health arising from systemic racism, which we were not able to represent in our analysis, may contribute to the disparities we observed.^40^ Our findings point to the need for improved ascertainment of geriatric impairments to identify functional deficits when treating women in marginalized groups. Health system-based interventions for Black women and other groups at risk for nonresilient trajectories, including targeted navigation, cancer prehabilitation, and supportive care during treatment, may improve the likelihood of functional recovery posttreatment.^41-44^

We also identified several aging-related factors that were associated with nonresilient frailty trajectories, including claims for home hospital beds, wheelchairs, oxygen, Parkinson’s disease, and older age. Other risk factors for frailty decline following chemotherapy initiation include inflammatory biomarkers, such as interleukin-6 (IL-6) and C-reactive protein (CRP).^13^ Findings from our study and others suggest that assessing a broad range of aging-related domains may help identify older women with breast cancer who are at the highest risk of decline during adjuvant chemotherapy treatment. The gold standard for evaluating aging-related domains in oncology care is the comprehensive geriatric assessment, which is currently recommended by the American Society of Clinical Oncology for all older adults with cancer initiating chemotherapy.^45^ Prior prospective studies have demonstrated that geriatric assessment-driven interventions can reduce cancer treatment-related toxicities and improve outcomes in older adults with cancer.^45^

Our study serves as an example of how claims-based frailty measures may be used to conduct patient-centered geriatric oncology research. Frailty is an important prognostic indicator that should be considered when making treatment decisions for older adults with cancer. However, many cancer treatments may lead to changes in physical functioning for older adults and longitudinal assessments of frailty are not standard practice in oncology care. Preservation of independence and physical functioning are important endpoints from the perspectives of older adults with cancer and are closely linked to quality of life.^46,47^ While clinical trials are the gold standard for measuring the effects of pharmacological treatments on cancer outcomes, older adults with cancer have historically been underrepresented in clinical trials.^48,49^ In addition, many clinical trials do not capture information on important patient-centered endpoints such as frailty and physiological functioning. Future research can leverage SEER-Medicare data, which is readily available to researchers through data use agreements, to study changes in frailty as an endpoint.

Strengths of our study include the use of the SEER-Medicare database, which includes a large and diverse sample of older adults with incident cancer and a noncancer sample. Because of the large sample, we were able to identify claims-based frailty trajectories with low prevalence, as they still represented hundreds of women in the SEER-Medicare database. Identifying these groups in smaller prospective observational studies and clinical trials would be near impossible, even with frequent frailty assessments. Another strength of our study is the use of epidemiologic methods, including SMR and inverse probability of attrition weighting, to account for differences in covariate distributions and informative attrition in our study populations. These methods are especially important in studies of older adults, where death and attrition during follow-up are common.^50-52^

Our results should be interpreted considering their limitations. First, our restriction to women receiving adjuvant chemotherapy implicitly leads to the selection of a healthier group of women with breast cancer. Our findings likely do not generalize to the sickest or frailest women with breast cancer who would be deemed unfit for surgery and chemotherapy by their physicians. This restriction may partially explain why the average claims-based frailty score was higher among women receiving chemotherapy than the comparator cohort without cancer. Second, some of the changes in frailty we observed following chemotherapy initiation may be due to the effects of surgery rather than chemotherapy. Third, we did not evaluate chemotherapy duration and some women in our cohort may have discontinued treatment before completing a full course. Fourth, we restricted our sample to women since risk factors for frailty differ by gender^53,54^ and our results may not generalize to men with breast cancer or people with other types of cancer. Finally, some of the changes we observed in the claims-based frailty score may reflect changes in services received during cancer treatment rather than true changes in frailty. It is possible that deferral of routine primary care in the face of competing priorities from a cancer diagnosis and treatment, rather than a true change in frailty, might explain some of our findings. We tested this in 2 sensitivity analyses related to cancer and lipid screening services and found similar results as in the primary analysis, which suggests that not all the patterns we observed can be explained by changes in health care services. Future studies with other data linkages may help provide additional support regarding whether the changes we observed were clinically meaningful and identify modifiable factors that enhance the resilient trajectory after chemotherapy.

## Conclusion

Using the SEER-Medicare database, we described longitudinal claims-based frailty trajectories and identified predictors of nonresilience in women with early-stage breast cancer undergoing adjuvant chemotherapy. Our study demonstrates the feasibility of using claims-based frailty indices to assess changes in frailty during cancer treatment and can be expanded to investigate application to other cancer types.

## Supplementary material

Supplementary material is available at *The Oncologist* online.

oyae092_suppl_Supplementary_Material

## Data Availability

This article used data from the Surveillance, Epidemiology, and End Results cancer registries with linkage to Medicare claims and enrollment data. Data are available to researchers through data use agreements: https://health caredelivery.cancer.gov/seermedicare/.
