# Supplementary material for: Longitudinal trajectories of a claims-based frailty measure during adjuvant chemotherapy in women with stage I-III breast cancer
Source: Oncologist. 2024 May 8;29(10):e1291–301. doi: 10.1093/oncolo/oyae092 (PMC11449071; doi:10.1093/oncolo/oyae092)
Supplement: oyae092_suppl_Supplementary_Material [file oyae092_suppl_supplementary_material.docx]

**Supplemental file**

**Supplemental Tables**

Supplemental Table 1 Codes to identify study variables

Supplemental Table 2 Prevalence of claims-based frailty indicators at the time of adjuvant chemotherapy initiation, 4 months post-initiation, and 10-months post-initiation in women with stage I-III breast cancer in the SEER-Medicare database

Supplemental Table 3 Baseline characteristics of women with stage I-III breast cancer receiving adjuvant chemotherapy in the SEER-Medicare database, stratified by claims-based frailty trajectory

**Supplemental Figures**

Supplemental Figure 1 Study schematic for K-means longitudinal clustering analysis

Supplemental Table 1. Codes to identify study variables

| Variable | Codes |
| --- | --- |
| Breast cancer | ICD-O-3: C500-C509 |
| Breast cancer surgery |  |
| Mastectomy | CPT: 19180, 19182, 19200, 19220, 19240, 19303, 19304, 19305, 19306, 19307  ICD-9-PCS: 85.41, 85.42, 85.43, 85.44, 85.45, 85.46, 85.47, 85.48  ICD-10-PCS: 07T50ZZ, 07T60ZZ, 07T70ZZ, 07T80ZZ, 07T90ZZ, 0HTT0ZZ, 0HTU0ZZ, 0HTV0ZZ |
| Breast conserving therapy | CPT: 19120, 19125, 19126, 19160, 19162, 19301, 19302  ICD-9-PCS: 85.2x, 85.20, 85.21, 85.22, 85.23  ICD-10-PCS: 0HBT0ZZ, 0HBT3ZZ, 0HBT7ZZ, 0HBT8ZZ, 0HBTXZZ, 0HBU0ZZ, 0HBU3ZZ, 0HBU7ZZ, 0HBU8ZZ, 0HBUXZZ, 0HBV0ZZ, 0HBV3ZZ, 0HBV7ZZ, 0HBV8ZZ, 0HBVXZZ |
| Chemotherapy | HCPCS: C9415, J9000, J9001, J9002, Q2048, Q2049, Q2050, J9178, J9180, J9190, J8520, J8521, C9127, C9431, J9264, J9265, J9267, J9170, J9171, J9045, C9420, C9421, J8530, J9070, J9080, J9090, J9091, J9092, J9093, J9094, J9095, J9096, J9097, C9414, C9425, J8560, J9181, J9182, C9418, J9060, J9062, C9280, J9179, J9201, J9198, C9240, J9207, J8610, J9250, J9260, J9293, C9440, J9390, J9360, J8999, J9999 |
| Breast cancer screening ^a^ | ICD-9-CM: V76.1  ICD-10-CM: Z12.3  CPT: 76090, 76091, 76092, 77055  HCPCS: G0202, G0203 |
| Flu vaccine | HCPCS: 90653-90658, 90660-90664, 90666-90668, 90672-90674, 90682, 90686, 90688, 90694, 90674, 90756, 90689, Q2034-Q2039, G0008 |

Abbreviations: CPT=Current Procedural Terminology; HCPCS=Healthcare Common Procedure Coding System; ICD-9-CM=International Classification of Diseases, Ninth Revision, Clinical Modification; ICD-10-CM=International Classification of Diseases, Tenth Revision, Clinical Modification; NOS=not otherwise specified.

^a^ We did not include codes for diagnostic mammography (HCPCS: G0204-G0206).

Supplemental Table 2. Prevalence of claims-based frailty indicators at the time of adjuvant chemotherapy initiation, 4 months post-initiation, and 10-months post-initiation in women with stage I-III breast cancer in the SEER-Medicare database

|  | Prevalence, n (%) | | | | | |
| --- | --- | --- | --- | --- | --- | --- |
|  | Chemotherapy initiation | | 4 months post-initiation | | 10 months post-initiation | |
| Lipid abnormality | 13,916 | (64) | 11,713 | (55) | 10,985 | (52) |
| Cancer screening | 12,366 | (57) | 2,925 | (14) | 5,161 | (25) |
| Arthritis/joint conditions | 9,627 | (45) | 8,539 | (40) | 7,495 | (36) |
| Psychiatric diagnoses | 5,041 | (23) | 5,391 | (25) | 3,706 | (18) |
| Rehabilitation services | 3,650 | (17) | 4,405 | (21) | 4,413 | (21) |
| Heart failure | 3,431 | (16) | 4,052 | (19) | 2,743 | (13) |
| Vertigo | 1,414 | (7) | 1,706 | (8) | 1,510 | (7) |
| Podiatric care | 1,137 | (5) | 1,384 | (6) | 1,784 | (8) |
| Bladder dysfunction | 1,110 | (5) | 1,064 | (5) | 945 | (4) |
| Weakness | 959 | (4) | 1,597 | (7) | 1,419 | (7) |
| Ambulance/life support | 912 | (4) | 2,034 | (10) | 1,108 | (5) |
| Dementia | 745 | (3) | 791 | (4) | 759 | (4) |
| Stroke/brain injury | 645 | (3) | 827 | (4) | 685 | (3) |
| Home oxygen | 482 | (2) | 646 | (3) | 642 | (3) |
| Hypotension/shock | 436 | (2) | 1,433 | (7) | 581 | (3) |
| Skin ulcer (decubitus) | 322 | (1) | 556 | (3) | 470 | (2) |
| Paralysis | 181 | (1) | 201 | (1) | 157 | (1) |
| Wheelchair | 141 | (1) | 247 | (1) | 323 | (2) |
| Parkinson' disease | 113 | (1) | 116 | (1) | 127 | (1) |
| Home hospital bed | 80 | (<1) | 119 | (1) | 151 | (1) |

Abbreviations: SEER=Surveillance, Epidemiology, and End Results.

Supplemental Table 3. Baseline characteristics of women with stage I-III breast cancer receiving adjuvant chemotherapy in the SEER-Medicare database, stratified by claims-based frailty trajectory

| Characteristic | Robust  N=16,667 | | Resilient low/medium  N=3,214 | | Resilient medium/high  N=261 | | Non-resilient low-to-medium  N=749 | | Non-resilient low-to-high  N=222 | | Non-resilient high  N=135 | |
| --- | --- | --- | --- | --- | --- | --- | --- | --- | --- | --- | --- | --- |
| Age, median (IQR) | 70 | (67, 73) | 73 | (69, 78) | 74 | (70, 79) | 74 | (70, 79) | 74 | (70, 79) | 74 | (70, 78) |
| Race,^a^ n (%) |  |  |  |  |  |  |  |  |  |  |  |  |
| White | 15,027 | (90.2) | 2,344 | (72.9) | 197 | (75.4) | 566 | (75.6) | *** | | 94 | (69.6) |
| Black | 1,168 | (7.0) | 396 | (12.3) | 48 | (18.5) | 126 | (16.8) | 37 | (16.7) | 29 | (21.5) |
| Other | 473 | (2.8) | 474 | (14.8) | 16 | (6.2) | 57 | (7.6) | *** | | 12 | (8.9) |
| Hispanic ethnicity, n (%) | 1,090 | (6.5) | 86 | (2.7) | 16 | (6.1) | 41 | (5.5) | *** | | *** | |
| Census region, n (%) |  |  |  |  |  |  |  |  |  |  |  |  |
| Northeast | 3,353 | (20.1) | 555 | (17.3) | 41 | (15.7) | 118 | (15.8) | 45 | (20.3) | 18 | (13.3) |
| West | 6,891 | (41.3) | 1,406 | (43.7) | 110 | (42.1) | 313 | (41.8) | 86 | (38.7) | 47 | (34.8) |
| Midwest | 3,130 | (18.8) | 619 | (19.3) | 62 | (23.8) | 152 | (20.3) | 45 | (20.3) | 29 | (21.5) |
| South | 3,293 | (19.8) | 634 | (19.7) | 48 | (18.4) | 166 | (22.2) | 46 | (20.7) | 41 | (30.4) |
| Stage at diagnosis, n (%) |  |  |  |  |  |  |  |  |  |  |  |  |
| I | 3,967 | (23.8) | 589 | (18.3) | 38 | (14.6) | 114 | (15.2) | 30 | (13.5) | 18 | (13.3) |
| II | 9,025 | (54.1) | 1,686 | (52.5) | 135 | (51.7) | 384 | (51.3) | 113 | (50.9) | 62 | (45.9) |
| III | 3,675 | (22.0) | 939 | (29.2) | 88 | (33.7) | 251 | (33.5) | 79 | (35.6) | 55 | (40.7) |
| Type of surgery, n (%) |  |  |  |  |  |  |  |  |  |  |  |  |
| Mastectomy | 6,432 | (38.6) | 1,447 | (45.0) | 144 | (55.2) | 331 | (44.2) | 100 | (45.0) | 75 | (55.6) |
| BCT | 10,235 | (61.4) | 1,767 | (55.0) | 117 | (44.8) | 418 | (55.8) | 122 | (55.0) | 60 | (44.4) |
| T stage, n (%) |  |  |  |  |  |  |  |  |  |  |  |  |
| T1 | 7,355 | (44.1) | 1,133 | (35.3) | 80 | (30.8) | 227 | (30.3) | 67 | (30.2) | 32 | (23.9) |
| T2 | 7,761 | (46.7) | 1,647 | (51.4) | 143 | (55.0) | 401 | (53.5) | 126 | (56.8) | 67 | (50.0) |
| T3 | 1,110 | (6.7) | 270 | (8.4) | *** | | 81 | (10.8) | 16 | (7.2) | 24 | (17.9) |
| T4 | 416 | (2.5) | 158 | (4.9) | *** | | 40 | (5.3) | 13 | (5.9) | 11 | (8.2) |
| N stage, n (%) |  |  |  |  |  |  |  |  |  |  |  |  |
| N0 | 7,431 | (44.6) | 1,338 | (41.7) | 93 | (35.6) | 290 | (38.7) | 75 | (33.8) | 47 | (34.8) |
| N1 | 6,124 | (36.8) | 1,104 | (34.4) | 97 | (37.2) | 250 | (33.4) | 76 | (34.2) | 46 | (34.1) |
| N2 | 2,040 | (12.3) | 489 | (15.3) | 42 | (16.1) | 119 | (15.9) | 42 | (18.9) | 23 | (17.0) |
| N3 | 1,057 | (6.3) | 274 | (8.5) | 29 | (11.1) | 90 | (12.0) | 29 | (13.1) | 19 | (14.1) |
| Tumor grade, n (%) |  |  |  |  |  |  |  |  |  |  |  |  |
| Well or moderately differentiated | 8,286 | (49.7) | 1,339 | (41.7) | 107 | (41.0) | 282 | (37.6) | 88 | (39.4) | 52 | (38.2) |
| Poorly or undifferentiated | 8,381 | (50.3) | 1,875 | (58.3) | 154 | (59.0) | 467 | (62.4) | 134 | (60.6) | 83 | (61.8) |
| Subtype,^b^ n (%) |  |  |  |  |  |  |  |  |  |  |  |  |
| HR+/HER2+ | 1,705 | (17.4) | 393 | (20.2) | 25 | (16.3) | 80 | (17.9) | *** | | 18 | (24.3) |
| HR+/HER2- | 5,256 | (53.8) | 866 | (44.6) | 67 | (43.8) | 196 | (43.8) | 55 | (47.4) | 33 | (44.6) |
| HR-/HER2+ | 671 | (6.9) | 195 | (10.0) | 12 | (7.8) | 63 | (14.1) | *** | | 11 | (14.9) |
| Triple negative | 2,141 | (21.9) | 489 | (25.2) | 49 | (32.0) | 109 | (24.3) | 34 | (29.3) | 12 | (16.2) |
| Gagne combined comorbidity score,^b^ n (%) |  |  |  |  |  |  |  |  |  |  |  |  |
| ≤0 | 13,045 | (78.3) | 1,899 | (59.1) | 95 | (36.4) | 360 | (48.1) | 112 | (50.5) | 55 | (40.7) |
| 1 | 2,199 | (13.2) | 570 | (17.7) | 40 | (15.3) | 128 | (17.1) | 35 | (15.8) | 23 | (17.0) |
| 2 | 776 | (4.7) | 316 | (9.8) | 25 | (9.6) | 97 | (13.0) | 22 | (9.9) | 14 | (10.4) |
| ≥3 | 647 | (3.9) | 429 | (13.3) | 101 | (38.7) | 164 | (21.9) | 53 | (23.9) | 43 | (31.9) |
| Pre-index flu vaccine, n (%) | 5,120 | (30.7) | 945 | (29.4) | 74 | (28.4) | 217 | (29.0) | 63 | (28.4) | 35 | (25.9) |

***Suppressed due to sample size reporting restrictions. The SEER-Medicare data use agreement does not allow publication of cell sizes less than 11. Abbreviations: BCT=breast conserving therapy; HER2=human epidermal growth factor receptor 2; HR=hormone receptor; IQR=interquartile range.

^a^ Other category includes Alaskan Native/American Indian and Asian and Pacific Islander.

^b^ Subtype is not reliably captured in SEER-Medicare until 2010. Proportions reflect the frequency for individuals diagnosed in 2010 or onwards.

Supplemental Figure 1. Study schematic for K-means longitudinal clustering analysis


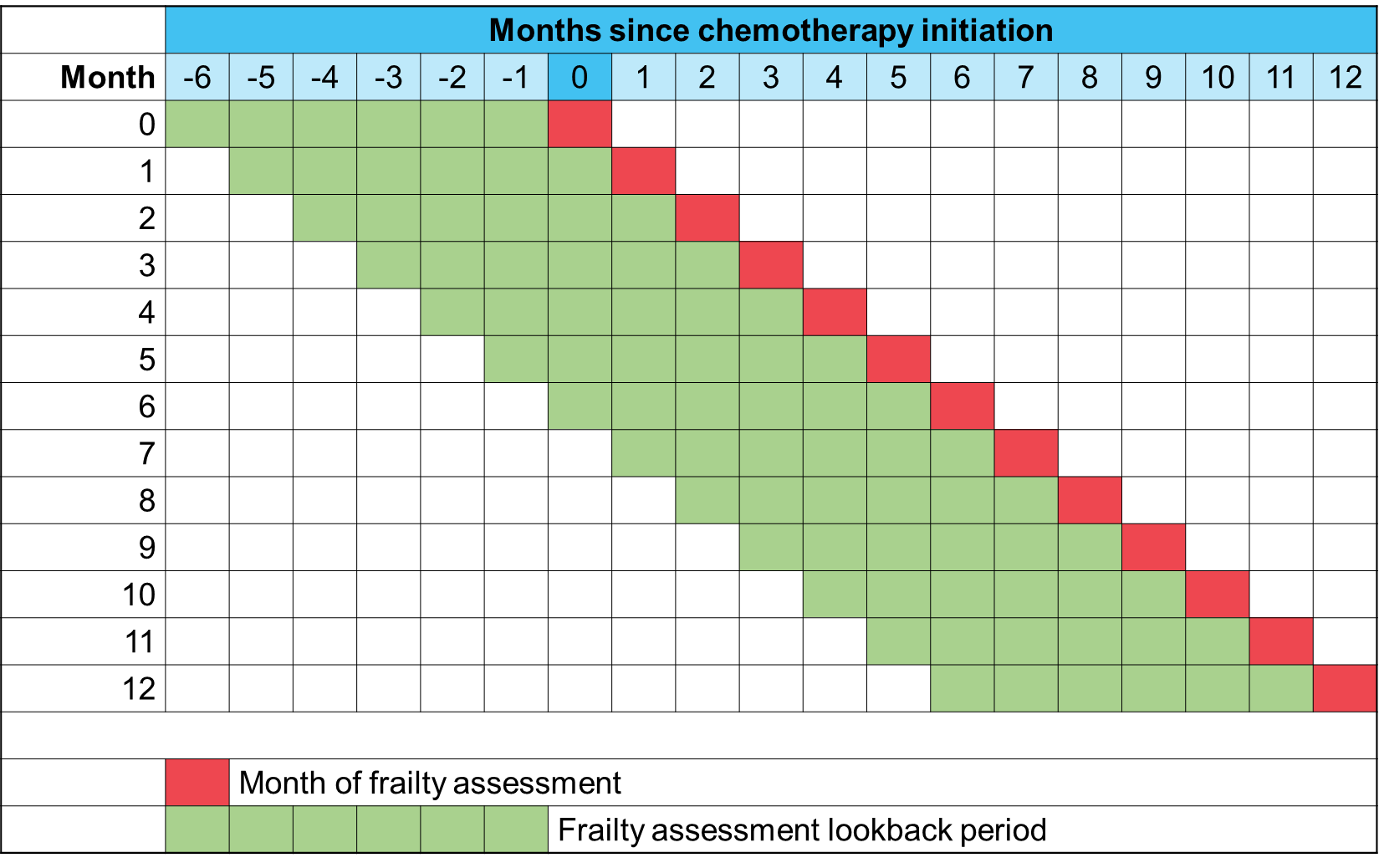


Notes: This study schematic displays the design used to estimate the predicted probability of frailty using the Faurot frailty index each month from chemotherapy initiation for up to 12 months. The Faurot frailty index was re-calculated each month using claims during the 180 days (6 months) prior to the month.
